# Subhealth: definition, criteria for diagnosis and potential prevalence in the central region of China

**DOI:** 10.1186/1471-2458-13-446

**Published:** 2013-05-04

**Authors:** Guolin Li, Fuxia Xie, Siyu Yan, Xiaofei Hu, Bo Jin, Jun Wang, Jinfeng Wu, Dazhong Yin, Qingji Xie

**Affiliations:** 1 The Key Laboratory of Protein Chemistry and Developmental Biology of Ministry of Education, College of Life Sciences, Hunan Normal University, Changsha, Hunan 410081, P. R. China; 2 Qingyuan City People’s Hospital of Jinan University, Qingyuan, Guangdong 511518, P. R. China; 3 The Key Laboratory of Chemical Biology and Traditional Chinese Medicine Research of Ministry of Education, College of Chemistry and Chemical Engineering, Hunan Normal University, Changsha, Hunan 410081, P. R. China; 4 The Fourth Hospital of Changsha, Affiliated Hospital of Hunan Normal University, Changsha, Hunan 410081, P.R. China

**Keywords:** Subhealth, Health, Disease, Oxidative stress, Thiobarbituric acid-reactive substances (TBARS)

## Abstract

**Background:**

A full evaluation of health conditions is necessary for the effective implementation of public health interventions. However, terms to address the intermediate state between health and disease are lacking, leading the public to overlook this state and thus increasing the risks of developing disease.

**Methods:**

A cross-sectional health survey of 1,473 randomly recruited Chinese Han adults of both sexes living in the central region of China. The criteria for diagnosis of subhealth was defined as the presence of ≥ 1 of the following abnormalities: body mass index ≥ 25 kg/m^2^ or waist circumference ≥ 102 cm in men and 88 cm in women; systolic pressure 120–139 mmHg and/or diastolic pressure 80–89 mmHg; serum triglyceride level ≥ 150 mg/dL and/or total cholesterol level ≥ 200 mg/dL and/or high-density lipoprotein cholesterol level < 40 mg/dL in men and 50 mg/dL in women; serum glucose level 110–125 mg/dL; estimated glomerular filtration rate 60–89 ml/min/1.73 m^2^; levels of liver enzymes in liver function tests between 41–59 U/L, or with fatty liver disease but < 33% of affected hepatocytes; levels of oxidative stress biomarkers beyond the reference range of 95%; or problems with both sleep quality and psychological state.

**Results:**

The prevalences of subhealth and disease in the central region of China were 36.6% and 43.1%, respectively. The prevalence of disease increased from 26.3% in participants aged 20–39 years, to 47.6% and 78.9% for participants aged 40–59 years and those aged 60 years or older, respectively. Compared with participants aged 20–39, the prevalences of health and subhealth in participants aged 60 years or older decreased by 86.7% and 60.3%, respectively. The prevalence of subhealth was increased in association with increases in lifestyle risk scores, while the prevalences of both health and disease were reduced.

**Conclusion:**

The prevalences of subhealth and disease are high in central China. Subhealth is associated with high lifestyle risk scores. Both the health care sector and the public should pay more attention to subhealth. Lifestyle modifications and/or psychological interventions are needed to ameliorate these conditions.

## Background

A full evaluation of health conditions is necessary for the effective implementation of public health interventions. However, terms to address the intermediate state between health and disease are lacking, leading the public to overlook this state, and thus increasing the risks of developing disease. We therefore coined the term ‘subhealth’ by added the prefix ‘sub’ meaning ‘beneath’ to health, to represent pre-disease condition. This was defined as a state characterized by some disturbances in psychological behaviors or physical characteristics, or in some indices of medical examination, with no typical pathologic features.

Although metabolic syndrome, which is a combination of disturbed fasting glucose, fat accumulation and distribution, blood pressure, and blood lipids, meets some characteristics of subhealth, it mainly represents a collection of risk factors for developing type 2 diabetes mellitus and cardiovascular diseases [1-5]. Moreover, even regardless of various diagnostic criteria for metabolic syndrome have been proposed by different organizations [2,3,6-10], the metabolic syndrome as an entity might only be of conceptual value, and cardiovascular risk associated with the syndrome seems to be no greater than sum of its components [11,12]. A recent longitudinal cohort study in non-diabetic individuals has also illustrated that fasting plasma glucose levels are as good as or potentially better than metabolic syndrome for predicting diabetes [13]. Furthermore, the inclusive and exclusive criteria for metabolic syndrome are imperfect, with little or no accounting for psychological behaviors and physical characteristics, and with the inclusion of some diseases, such as hypertension and diabetes [8,12,14]. Thus the importance of metabolic syndrome for estimating public health and general health care may be limited.

Considering that allostasis, or homeostasis, is the basis of all physiological and psychological activities [15,16], allostatic load or stress, should be a common process behind all-cause diseases, and a potential index of overall health conditions. Allostatic load or stress occurs when allostasis is targeted by stressful stimuli, and the normal psychological and/or physiological states, and even health conditions, are threatened [15,17]. Metabolic syndrome may represent stress largely related to the stressful stimuli of the “coach potato” lifestyle (high calorie intake and physical inactivity). However, stresses induced by other stimuli would also be expected to influence health. Because stress represents disturbed allostasis, a subsequent energy-consuming recovery process is likely to be recruited by several biological systems, leading to enhanced production of reactive oxygen species and consequent oxidative stress. This suggests that oxidative stress, a potential index of general stress, might be regarded as one component of subhealth. Accumulating data indicate an essential role for oxidative stress in the mechanisms underlying the development and progression of metabolic syndrome [18,19] and even aging and age-related diseases [20,21]. To the best of our knowledge, no reports have included oxidative stress in assessments of the general public health state.

By combining general stress, psychological behaviors, physical characteristics and some indices of medical examinations, we sought to establish criteria for the diagnosis of subhealth, and to estimate the potential prevalence of subhealth in the central region of China. Subhealth was evaluated in volunteers by measuring oxidative stress, indicated by plasma levels of thiobarbituric acid-reactive substances (TBARS), and by using questionnaires and physical examinations.

## Methods

### Chemicals

The chemicals 1,1,3,3-tetramethoxypropane and thiobarbituric acid were purchased from Sigma-Aldrich (St. Louis, MO, USA). Other chemicals (analytical grade) were from Sangon Biotech Co. Ltd. (Shanghai, China). Water was produced using a Milli-Q Plus purification system (Millipore, Bedford, MA).

### Participants and questionnaire

The study was approved by the Ethics Committee/Institutional Review Board of Hunan Normal University. All study volunteers signed informed consent forms before their inclusion in the project. From September 2009 to August 2011, a consecutive unselected recruitment was applied to recruit the subjects from those attending our institution. The total study subjects consisted of 1615 Chinese Han adults of both genders living in the urban area of Changsha City in the Central Region of China. After excluded the subjects missing baseline data, 1473 adults were included in analysis. The basic characteristics of the participants are shown in Table 1.

**Table 1:** **Basic characteristics of the 1473 Chinese adult participants**^**§**^

**Factors**	**All (N =1473)**	**Health (N = 299)**	**Subhealth (N = 539)**	**Disease (N = 635)**
Age, mean (SD), years	44.27 (14.52)	36.38 (10.92)	40.98 (12.11) ^**^	50.67 (15.14) ^**††^
Gender, male, No%	993 (67.41)	164 (55.07)	370 (68.83)	459 (72.53)
Blood pressure, mean (SD) ,mmHg				
Systolic	123.14(15.92)	108.64 (8.42)	119.45 (11.22) ^**^	130.79 (16.92) ^**††^
Diastolic	75.05 (11.46)	65.77 (6.30)	72.75 (8.67) ^**^	79.93 (12.43) ^**††^
Blood lipids, mean (SD), mmol/L				
Total cholesterol	4.40 (0.84)	3.94 (0.77)	4.39 (0.84) ^**^	4.54 (0.84) ^**†^
Triglyceride	1.64 (1.27)	0.93 (0.60)	1.53 (1.00) ^**^	1.96 (1.53) ^**††^
FBG, mean (SD), mmol/L	5.03 (1.26)	4.66 (0.47)	4.77 (0.53) ^**^	5.36 (1.70) ^**††^
BMI, mean (SD), kg/m^2^	23.71 (3.22)	21.06 (2.07)	23.40 (2.82) ^**^	24.80 (3.31) ^**††^
Creatinine, mean (SD), μmol/L	84.72 (16.54)	72.12 (12.66)	84.63 (15.30) ^**^	88.15 (16.86) ^**††^
eGFR, mean (SD), ml/min per 1.73m^2^	93.68 (17.66)	108.35 (8.96)	93.82 (15.29) ^**^	86.69 (18.37) ^**††^
Liver function, mean (SD) U/L				
ALT	27.99 (23.69)	21.22 (17.78)	26.21 (19.34) ^**^	31.88 (27.93) ^**††^
AST	22.95 (10.00)	19.91 (7.00)	22.20 (9.10) ^**^	24.64 (11.24) ^**††^
TBARS, mean (SD), μmol/L	3.72 (1.21)	3.31 (0.80)	3.71 (1.14) ^**^	3.92 (1.36) ^**†^
Sleep quality, well, No (%)	1001 (67.96)	215 (71.90)	341 (63.30)	445 (70.10)
Healthy diet intake, No (%)	791 (53. 70)	149 (50.00)	281 (52.10)	360 (56.71)
Current tobacco consumption, No (%)	584 (39.65)	91 (30.04)	235 (43.68)	258 (40.68)
Current alcohol intake, No (%)	804 (54.58)	141 (47.36)	303 (56.02)	360 (56.70)
Physical activity level, >1h/w, No (%)	427 (28.99)	64 (21.18)	143 (26.50)	220 (34.64)
LRS, mean (SD)	2.28 (1.23)	2.10 (1.21)	2.44 (1.26) ^**^	2.24 (1.22) ^††^

^§^The 1473 Chinese Han adult participants were recruited from the urban area of Changsha City in the Central Region of China from September 2009 to August 2011, aged 20–91 and both genders.

^**^P<0.01 compared to healthy subjects; ^†^P<0.05 compared to subhealthy subjects; ^††^P<0.01 compared to subhealthy subjects.

Abbreviations: FBG, fasting blood glucose; BMI, body mass index; eGFR, estimated glomerular filtration rate; ALT, alanine aminotransferase; AST, aspartate aminotransferase; TBARS, thiobarbituric acid-reactive substances; LRS, lifestyle risk scores.

The questionnaire recorded information on demographics (age and gender), lifestyle habits (sleep, diet, smoking history, drinking history and physical activity), detailed medical history, and psychological state. The questionnaire included five possible answers for sleep quality: well, insomnia, dreaminess, restless sleep, and other problems. There were six possible options for psychological state: well, fugue, anxiety, nervousness, agitation, and depression. Subjects whose answers contained at least one ‘well’ for sleep quality or psychological state were not classified as suffering from mental subhealth.

### Lifestyle risk score

As shown in Table 2, the lifestyle risk score (LRS) was calculated by summing the scores from five lifestyle factors: diet intake, sleep quality, tobacco consumption, alcohol intake, and physical activity level. Each was scored on the basis of assigning 0 for healthy, or 1 for unhealthy. Each subject was thus assigned a score of 0–5, where a higher score implied an unhealthier lifestyle.

**Table 2:** **Lifestyle risk scores**^**§**^

**Lifestyles**	**Assigned scores**	
	**1**	**0**
Diet intake	unhealthy	healthy
Sleep quality	not too well	well
Tobacco consumption	yes	no
Alcohol intake	yes	no
Physical activity level	≤1h/w	>1h/w

^§^The scores were applied to 1473 Chinese Han adult recruited from the urban area of Changsha City in the Central Region of China from September 2009 to August 2011, aged 20–91 and both genders.

### Blood sampling

After overnight fasting (12 hours), blood samples (5 mL) from the median cubital vein on the inside of the elbow were collected into vacutainer tubes containing ethylene diamine tetra-acetic acid, according to standard blood collection procedures [22], and stored at 0–4°C. All analyses were carried out within 8 h of sampling.

### Physical examination

Stature, body weight and body mass index (BMI) were detected using an ultrasonic body scale (SK-CK; Sonka Electronic Technologies Co. Ltd., Shenzhen, China). The BMI cut-off point for overweight was 25 kg/m^2^, as advocated by the World Health Organization (WHO) [23].

After resting for 30 min, each participant’s blood pressure was measured three times in the sitting position, with the right arm relaxed and well supported by a table, at an angle of 45° from the trunk, using an automatic electronic sphygmomanometer (Ken2-BPMSP-1; Pengcheng Healthcare Products Co. Ltd., Shenzhen, China). According to the suggestion of the American Heart Association [24], subjects whose mean systolic/diastolic blood pressures were ≥ 140 mmHg/90 mmHg, or who were receiving antihypertensive medication were classified as hypertensive, those with blood pressure of 120–139 mmHg/80–89 mmHg but who had never been told that they had high blood pressure were defined as prehypertensives, and those with blood pressure of 90–119 mmHg/60–79 mmHg were classified as normotensive.

Blood lipids (serum total cholesterol and triglycerides), fasting plasma glucose, renal function (serum creatinine) and liver function (aspartate transaminase [AST], and alanine transaminase [ALT]) were measured using a chromatographic enzymatic method with a Mindray BS-40 automatic analyzer (Mindray Co. Ltd., Shenzhen, China). Serum hepatitis B viral antigens and antibodies were detected using a microplate reader (MR-96; Mindray Co. Ltd.). The diagnosis of fatty liver disease (FLD) was based on ultrasound examination using a Mindray DP-9900 Plus Digital B/W Ultrasound System (Mindray Co. Ltd.).

Serum total cholesterol and serum triglycerides were categorized as normal, borderline high, or high, based on the Third Report of the National Cholesterol Education Program Expert Panel on Detection, Evaluation, and Treatment of High Blood Cholesterol in Adults (Adult Treatment Panel III) final report [10]. Fasting blood glucose was classified as normal (< 6.0 mM), pre-diabetic (6.1–6.9 mM), or diabetic (> 7.0 mM), on the basis of the WHO criteria [2,25].

The estimated glomerular filtration rate (eGFR) was calculated from serum creatinine using the Chronic Kidney Disease Epidemiology Collaboration (CKD-EPI) formula [26]. Based on eGFR, renal function was categorized according to the related classifications [27,28]: health (eGFR ≥ 90 ml/min/1.73 m^2^ and no proteinuria), subhealth (60 ≤ eGFR < 90 ml/min/1.73 m^2^ and no proteinuria) and disease (eGFR < 60 ml/min/1.73 m^2^ or with proteinuria).

Hepatic health (all diagnostic indices of liver function, FLD and hepatitis B were normal), subhealth (mild FLD or slightly elevated levels of ALT or AST) and disease (hepatitis, severe FLD, high levels of ALT or AST, or both mild FLD and high levels of ALT or AST) were grouped on the basis of liver function tests, FLD and hepatitis B, and related reference ranges or guidelines [29-31].

### TBARS assay

A modified thiobarbituric acid method [32] was used to determine the level of oxidative stress in plasma. Level of oxidative stress was expressed as TBARS determined using an LS-50B spectrofluorometer (Perkin-Elmer Corp., Norwalk, USA).

### Criteria for diagnosis of subhealth

According to the definition of subhealth, subjects with mental subhealth, overweight, prehypertension, pre-diabetes, serum blood lipids (triglycerides or total cholesterol) above the borderline high level, renal subhealth, hepatic subhealth or TBARS ≥ 5.09 μmol/L (based on the reference range of 95%) were categorized as subhealthy. In detail, the criteria for diagnosis of subhealth was defined as the presence of ≥ 1 of the following abnormalities, in the absence of disease characteristics: body mass index ≥ 25 kg/m^2^ or waist circumference ≥ 102 cm in men and 88 cm in women; systolic pressure 120–139 mmHg and/or diastolic pressure 80–89 mmHg; serum triglyceride level ≥ 150 mg/dL (1.69 mmol/L) and/or total cholesterol level ≥ 200 mg/dL (5.17 mmol/L) and/or high-density lipoprotein cholesterol level < 40 mg/dL (1.04 mmol/L) in men and 50 mg/dL (1.29 mmol/L) in women; serum glucose level 110–125 mg/dL (6.1–6.9 mmol/L); estimated glomerular filtration rate 60–89 ml/min/1.73 m^2^; levels of liver enzymes in liver function tests between the upper limit of normal and 50% above the upper limit of normal (41–59 U/L), or with fatty liver disease but < 33% of affected hepatocytes; levels of oxidative stress biomarkers beyond the reference range of 95%; or problems with both sleep quality and psychological state. Those self-reported or diagnosed with any disease, or with symptoms of hypertension or diabetes, were defined as diseased, and those with no sign of subhealth or disease were classified as healthy (Table 3).

**Table 3:** **Criteria for diagnosis of subhealth and disease**^**§**^

**Factors**	**Subhealth**	**Disease**
Blood pressure (mmHg)		
Systolic pressure	120-139	≥ 140
Diastolic pressure	80-89	≥ 90
Fasting blood glucose (mmol/L)	6.1-6.9	≥ 7.0
Blood lipids (mmol/L)		
Triacylglycerol	≥ 1.69	
Total cholesterol	≥ 5.17	
BMI (kg/m^2^)	≥25	
TBARS (μmol/L)	≥ 5.09	
Liver		
Liver function (U/L)		
ALT	41-59 and without FLD	≥ 60
AST	41-59 and without FLD	≥ 60
FLD (percentage of affected hepatocytes )	<33% and normal liver function	≥ 33%, or <33% and abnormal liver function
Hepatitis B		
HBsAg		positive
HBsAb		-
HBeAg		positive
HBeAb		positive
HBcAb		positive
HBcAb-IgM		positive
Renal function (ml/min per 1.73m^2^)		
eGFR	60-89	< 60
Mental state		
Sleep quality	Both have some problems	
Psychological state		

^§^The criteria were applied to 1473 Chinese Han adult recruited from the urban area of Changsha City in the Central Region of China from September 2009 to August 2011, aged 20–91 and both genders.

Abbreviations: BMI, body mass index; TBARS, thiobarbituric acid-reactive substances; ALT, alanine aminotransferase; AST: aspartate aminotransferase; FLD, fatty liver disease; eGFR, estimated glomerular filtration rate.

### Statistics

Results were presented as means ± standard deviation (SD). Statistical analysis of data was performed using predictive analytics software statistics 18.0 (SPSS Inc., Chicago, IL). The reference range of 95% for TBARS was calculated based on the TBARS dataset after removal of the outliers using Grubbs’ test at α = 0.05 [33]. The Kruskal-Wallis test was used for comparisons between groups. A *P* value < 0.05 was considered statistically significant.

## Results

The prevalences of subhealth and disease in the central region of China were 36.6% and 43.1%, respectively, while healthy participants accounted for only 20.3% of the total population (Table 1). There were significant differences in age and most factors of physical examination among the healthy, subhealthy and diseased groups. Although no lifestyle factor was independently and significantly associated with health state, the LRS was significantly higher in subhealthy participants (Table 1). Futher results from different diagnostic criteria displayed that blood pressure, BMI and eGFR were main responsible for the high prevalence of subhealth (Table 4).

**Table 4:** **The prevalence of health, subhealth, disease by different factors**^**§**^

**Factors**	**Health**	**Subhealth**	**Disease**
Blood pressure, No (%)	828 (56.21)	455(30.89)	190 (12.90)
Fasting blood glucose, No (%)	1381 (93.75)	45 (3.05)	47 (3.19)
Blood lipids			
Total cholesterol, No (%)	1302 (88.39)	171(11.61)	-
Triglyceride, No (%)	1296 (87.98)	177(12.02)	-
BMI, No (%)	1107 (75.15)	366(24.85)	-
TBARS, No (%)	1399 (94.98)	74(5.02)	-
Liver function, No (%)	1101 (74.75)	217 (14.73)	155 (10.52)
eGFR, No (%)	951 (64.56)	406 (27.56)	116 (7.88)
Mental state, No (%)	1288 (87.44)	185 (12.56)	-
With other diseases	-	-	181(12.29)

^§^The subjects were 1473 Chinese Han adult recruited from the urban area of Changsha City in the Central Region of China from September 2009 to August 2011, aged 20–91 and both genders.

Abbreviations: BMI, body mass index; eGFR, estimated glomerular filtration rate; TBARS, thiobarbituric acid-reactive substances.

**Figure 1:**
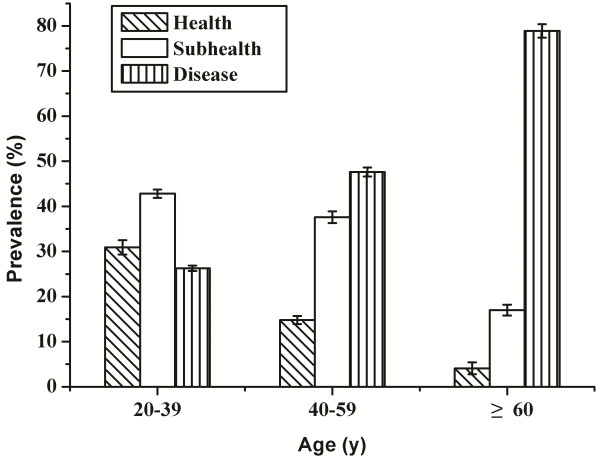
**Age-specific prevalence of health, subhealth and disease among 1473 Chinese adults.** The 1473 Chinese Han adult participants were recruited from the urban area of Changsha City in the Central Region of China from September 2009 to August 2011, aged 20–91 and both genders. Data were presented as percentage (SE).

**Figure 2:**
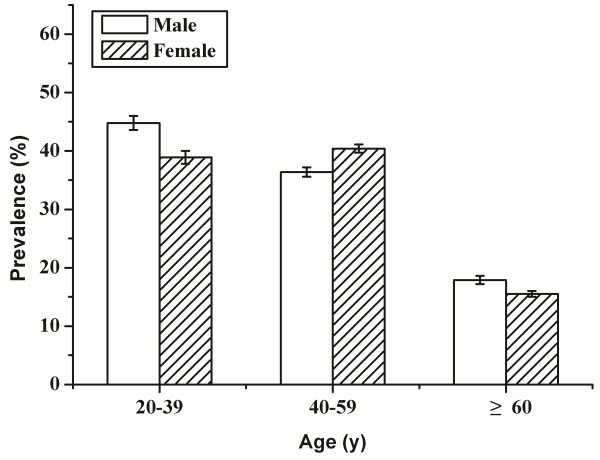
**Age-specific prevalence of subhealth by gender among 1473 Chinese adults.** The 1473 Chinese Han adult participants were recruited from the urban area of Changsha City in the Central Region of China from September 2009 to August 2011, aged 20–91 and both genders. Data were presented as percentage (SE).

The age-specific prevalences of health, subhealth and disease showed that the overall health state decreased gradually with increasing age (Figure 1). The prevalence of disease increased from 26.3% in participants aged 20–39 years, to 47.6% and 78.9% for participants aged 40–59 years and 60 years or older, respectively. The prevalences of both health and subhealth showed concomitant age-related decreases. Compared with participants aged 20–39, the prevalences of health and subhealth in participants aged 60 years or older decreased by 86.7% and 60.3%, respectively (Figure 1). There was no obvious difference in the prevalence of subhealth between males and females (Figure 2).

**Figure 3:**
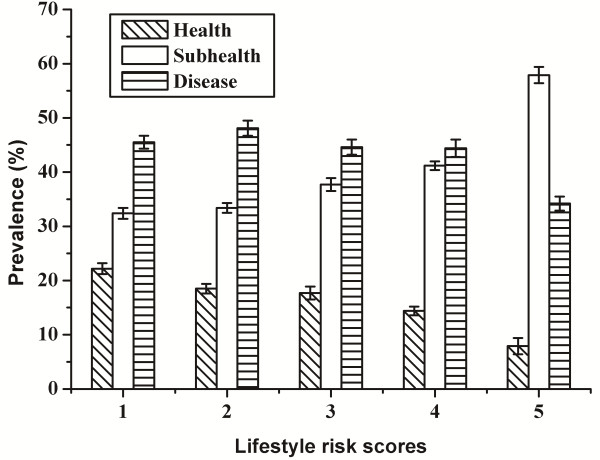
**The prevalence of health, subhealth, disease by LRS among 1473 Chinese adults.** The 1473 Chinese Han adult participants were recruited from the urban area of Changsha City in the Central Region of China from September 2009 to August 2011, aged 20–91 and both genders. Data were presented as percentage (SE).

The prevalences of health, subhealth, and disease by LRS were further analyzed. Intriguingly, only the prevalence of subhealth was obviously enhanced with increasing LRS, while the prevalences of both health and disease were reduced (Figure 3).

## Discussion

To our knowledge, this is the first definition for subhealth that fills the terminology gap between health and disease. This definition is necessary to draw people’s attention to their lifestyles, and to allow them to recover their physical and psychological states of health. If we regard health and disease as green and red traffic lights, respectively, then subhealth is the amber light. The absence of any light will increase the risk of a health-state traffic jam, as indicated by the results of this study. Subhealthy participants had the highest LRS, and only the prevalence of subhealth was positively associated with LRS. It is possible that, in the absence of any term defining the subhealthy condition, people fail to see the amber light and do not appreciate the need to make changes to their lifestyles or other factors.

We have therefore developed systematic diagnostic criteria for subhealth, based on the diagnostic criteria for metabolic syndrome [10,12,14], supplemented by measures of oxidative stress to represent general stress, combined sleep and psychological state for mental status, CKD-EPI-based eGFR for renal function, and liver related examinations for hepatic function state. These criteria, including general stress and physical and psychological conditions, provide information about the overall state of health. Moreover, a diagnosis is not difficult to achieve. It is possible to focus on an organ for organ-specific subhealth, or add an index of general stress and an easily-completed questionnaire and medical examination for overall subhealth.

The difference between subhealth and disease lies not only in the levels of the indices used, but more importantly in the means to recovery: generally, the cure of disease requires medication, while the threat to public health posed by subhealth can be eliminated simply by psychological interventions and/or lifestyle modifications. Several studies have suggested that physical activity, healthy diet and other lifestyle modifications can favorably affect components of subhealth [34-36]. However, subhealthy individuals are at high risk of developing disease if not treated in time.

Unhealthy lifestyles are associated with high risks of disease and mortality, while lifestyle improvements are associated with reductions in disease and all-cause mortality. Numerous studies have illustrated that healthy diet, moderate exercise, quitting cigarette smoking, and avoiding heavy drinking are separately associated with lower rates of death from all causes [37-42]. However, there are limited published studies reporting the relationship between combined lifestyle factors and mortality or diseases. By assigning a score of 1 for each poor lifestyle factor (unhealthy diet, sleep problems, smoking, drinking, and < 1 hour of physical activity per week) we calculated a combined LRS ranging from 0–5 for each subject. Based on this score, we demonstrated a positive effect of LRS on the prevalence of subhealth, and a negative effect on health and disease. It is possible that participants with disease, but not those defined as subhealthy, paid more attention to their lifestyles, which might explain why the correlations between mortality or disease and independent lifestyle factors was not always consistent.

In this cross-sectional study, we found approximately 80% of Chinese adults were subhealthy or diseased, based on our diagnostic criteria. This result is much higher than the reported prevalence of metabolic syndrome (23.7%), and even higher than the prevalence of one abnormal characteristic of metabolic syndrome (71.2%) reported in the Third National Health and Nutrition Examination Survey [43]. However, appropriate interventions could theoretically recover health from subhealth (with a prevalence of 36.6%). There is therefore an urgent need for the government, especially the health care sector, to make comprehensive efforts to lower the prevalence of subhealth. The cornerstone of intervention should be to reduce the LRS to zero.

The strengths of this study include the fact that it provides the first definition and criteria for the diagnosis of subhealth, making evaluation of the full state of health conditions feasible, and allowing the implementation of effective interventions. The application of LRS to investigate the combined impact of lifestyle factors on subhealth or disease is superior to assessing the independent impact of lifestyle factors. This cross-sectional study revealed a high prevalence of subhealth in the central region of China, which may serve prompt a major health care initiative aimed at treating subhealth, which has previously been ignored.

This study also had several limitations. Firstly, its sample was limited to Chinese Han adults from urban areas in the central region of China, and the applicability of the results to rural residents, and other nationalities and ethnic groups is unknown. Secondly, although TBARS is the most widely used biomarker of oxidative stress, other *in vivo* biomarkers exist, such as isoprostanes [44]. Finally, the method of evaluating mental conditions by a combination of sleep quality and psychological factors should not be absolute, but modify along with nationality, race and other characteristics of participants.

## Conclusion

This study provides the first definition and criteria for the diagnosis of subhealth, making evaluation of the full state of health conditions feasible, and allowing the implementation of effective interventions. The prevalences of subhealth and disease are high in central China. Subhealth is associated with high lifestyle risk scores. Both the health care sector and the public should pay more attention to subhealth. Lifestyle modifications and/or psychological interventions are needed to ameliorate these conditions.

## Competing interests

The authors declare that they have no competing interests.

## Authors’ contribution

The study was jointly designed by all authors. GL, FX, BJ, XH, JW, SY, JW, and QX were main responsible for data acquisition. GL, FX and SY were responsible for the data analysis and drafted the paper outline. GL, DY and QX were responsible for study supervision, ethical approvals and administrative, technical, or material support. Findings were jointly interpreted by all authors. All authors contributed to successive drafts. The final manuscript was approved by all authors.

## Pre-publication history

The pre-publication history for this paper can be accessed here:

http://www.biomedcentral.com/1471-2458/13/446/prepub

## References

[B1] Alberti KG Zimmet P Shaw J The metabolic syndrome–a new worldwide definition Lancet 2005 366 9491 1059 1062 10.1016/S0140-6736(05)67402-8 16182882

[B2] Alberti KG Zimmet PZ Definition, diagnosis and classification of diabetes mellitus and its complications. Part 1: diagnosis and classification of diabetes mellitus provisional report of a WHO consultation Diabet Med 1998 15 7 539 553 10.1002/(SICI)1096-9136(199807)15:7<539::AID-DIA668>3.0.CO;2-S 9686693

[B3] Grundy SM Brewer HB Jr Cleeman JI Smith SC Jr Lenfant C American Heart A National Heart L Blood I Definition of metabolic syndrome: report of the national heart, lung, and blood institute/american heart association conference on scientific issues related to definition Circulation 2004 109 3 433 438 10.1161/01.CIR.0000111245.75752.C6 14744958

[B4] Assmann G Guerra R Fox G Cullen P Schulte H Willett D Grundy SM Harmonizing the definition of the metabolic syndrome: comparison of the criteria of the adult treatment panel III and the international diabetes federation in united states american and european populations Am J Cardiol 2007 99 4 541 548 10.1016/j.amjcard.2006.08.045 17293200

[B5] Go AS Mozaffarian D Roger VL Benjamin EJ Berry JD Borden WB Bravata DM Dai S Ford ES Fox CS Heart disease and stroke statistics–2013 update: a report from the American heart association Circulation 2013 127 1 e6 e245 10.1161/CIR.0b013e31828124ad 23239837 PMC5408511

[B6] Alberti KG Zimmet P Shaw J Metabolic syndrome–a new world-wide definition. A consensus statement from the international diabetes federation Diabet Med 2006 23 5 469 480 10.1111/j.1464-5491.2006.01858.x 16681555

[B7] Balkau B Charles MA Drivsholm T Borch-Johnsen K Wareham N Yudkin JS Morris R Zavaroni I van Dam R Feskins E Frequency of the WHO metabolic syndrome in European cohorts, and an alternative definition of an insulin resistance syndrome Diabetes Metab 2002 28 5 364 376 12461473

[B8] Expert Panel on Detection E, Treatment of High Blood Cholesterol in A Executive summary of the third report of the national cholesterol education program (NCEP) expert panel on detection, evaluation, and treatment of high blood cholesterol in adults (adult treatment panel III) JAMA 2001 285 19 2486 2497 10.1001/jama.285.19.2486 11368702

[B9] Einhorn D Reaven GM Cobin RH Ford E Ganda OP Handelsman Y Hellman R Jellinger PS Kendall D Krauss RM American College of Endocrinology position statement on the insulin resistance syndrome Endocr Pract 2003 9 3 237 252 12924350

[B10] Third Report of the National Cholesterol Education Program (NCEP) Expert panel on detection, evaluation, and treatment of high blood cholesterol in adults (adult treatment panel III) final report Circulation 2002 106 25 3143 3421 12485966

[B11] Kahn R Metabolic syndrome–what is the clinical usefulness? Lancet 2008 371 9628 1892 1893 10.1016/S0140-6736(08)60731-X 18501420

[B12] Kahn R Buse J Ferrannini E Stern M American Diabetes A European Association for the Study of D The metabolic syndrome: time for a critical appraisal: joint statement from the american diabetes association and the european association for the study of diabetes Diabetes Care 2005 28 9 2289 2304 10.2337/diacare.28.9.2289 16123508

[B13] Sattar N McConnachie A Shaper AG Blauw GJ Buckley BM de Craen AJ Ford I Forouhi NG Freeman DJ Jukema JW Can metabolic syndrome usefully predict cardiovascular disease and diabetes? Outcome data from two prospective studies Lancet 2008 371 9628 1927 1935 10.1016/S0140-6736(08)60602-9 18501419

[B14] Alberti KG Eckel RH Grundy SM Zimmet PZ Cleeman JI Donato KA Fruchart JC James WP Loria CM Smith SC Jr Harmonizing the metabolic syndrome: a joint interim statement of the international diabetes federation task force on epidemiology and prevention; national heart, lung, and blood institute; american heart association; world heart federation; international atherosclerosis society; and international association for the study of obesity Circulation 2009 120 16 1640 1645 10.1161/CIRCULATIONAHA.109.192644 19805654

[B15] Li G He H Hormesis, allostatic buffering capacity and physiological mechanism of physical activity: a new theoretic framework Med Hypotheses 2009 72 5 527 532 10.1016/j.mehy.2008.12.037 19211194

[B16] McEwen BS Physiology and neurobiology of stress and adaptation: central role of the brain Physiol Rev 2007 87 3 873 904 10.1152/physrev.00041.2006 17615391

[B17] McEwen BS Protective and damaging effects of stress mediators N Engl J Med 1998 338 3 171 179 10.1056/NEJM199801153380307 9428819

[B18] Hampton T Mitochondrial defects may play role in the metabolic syndrome JAMA 2004 292 23 2823 2824 10.1001/jama.292.23.2823 15598899

[B19] Holvoet P Lee DH Steffes M Gross M Jacobs DR Jr Association between circulating oxidized low-density lipoprotein and incidence of the metabolic syndrome JAMA 2008 299 19 2287 2293 10.1001/jama.299.19.2287 18492970 PMC2562739

[B20] Beckman KB Ames BN The free radical theory of aging matures Physiol Rev 1998 78 2 547 581 9562038 10.1152/physrev.1998.78.2.547

[B21] Yin DZ Chen KJ The essential mechanisms of aging: Irreparable damage accumulation of biochemical side-reactions Exp Gerontol 2005 40 6 455 465 10.1016/j.exger.2005.03.012 15935593

[B22] Turgeon ML Turgeon ML Principles of blood collection Clinical hematology: theory and procedures 2004 4 Philadelphia: Lippincott Williams & Wilkins 18 40

[B23] WHO-Expert-Consultation Appropriate body-mass index for Asian populations and its implications for policy and intervention strategies Lancet 2004 363 9403 157 163 14726171 10.1016/S0140-6736(03)15268-3

[B24] Chobanian AV Bakris GL Black HR Cushman WC Green LA Izzo JL Jr Jones DW Materson BJ Oparil S Wright JT Jr The seventh report of the joint national committee on prevention, detection, evaluation, and treatment of high blood pressure: the JNC 7 report JAMA 2003 289 19 2560 2572 10.1001/jama.289.19.2560 12748199

[B25] Barr EL Zimmet PZ Welborn TA Jolley D Magliano DJ Dunstan DW Cameron AJ Dwyer T Taylor HR Tonkin AM Risk of cardiovascular and all-cause mortality in individuals with diabetes mellitus, impaired fasting glucose, and impaired glucose tolerance: the australian diabetes, obesity, and lifestyle study (AusDiab) Circulation 2007 116 2 151 157 10.1161/CIRCULATIONAHA.106.685628 17576864

[B26] Levey AS Stevens LA Schmid CH Zhang YL Castro AF 3rd Feldman HI Kusek JW Eggers P Van Lente F Greene T A new equation to estimate glomerular filtration rate Ann Intern Med 2009 150 9 604 612 10.7326/0003-4819-150-9-200905050-00006 19414839 PMC2763564

[B27] National Kidney Foundation K/DOQI clinical practice guidelines for chronic kidney disease: evaluation, classification, and stratification Am J Kidney Dis 2002 39 2 Suppl 1 S1 266 11904577

[B28] Finney H Newman DJ Price CP Adult reference ranges for serum cystatin C, creatinine and predicted creatinine clearance Ann Clin Biochem 2000 37 Pt 1 49 59 10672373 10.1258/0004563001901524

[B29] Angulo P Nonalcoholic fatty liver disease N Engl J Med 2002 346 16 1221 1231 10.1056/NEJMra011775 11961152

[B30] Lok AS McMahon BJ Chronic hepatitis B Hepatology 2007 45 2 507 539 10.1002/hep.21513 17256718

[B31] Siest G Schiele F Galteau M-M Panek E Steinmetz J Fagnani F Gueguen R Aspartate aminotransferase and alanine aminotransferase activities in plasma: statistical distributions, individual variations, and reference values Clin Chem 1975 21 8 1077 1087 1137913

[B32] Li G He H Yan H Zhao Q Yin D Does carbonyl stress cause increased blood viscosity during storage? Clin Hemorheol Microcirc 2010 44 2 145 154 20203369 10.3233/CH-2010-1263

[B33] Grubbs F Procedures for detecting outlying observations in samples Technometrics 1969 11 1 1 21 10.1080/00401706.1969.10490657

[B34] Bianchi C Penno G Romero F Del Prato S Miccoli R Treating the metabolic syndrome Expert Rev Cardiovasc Ther 2007 5 3 491 506 10.1586/14779072.5.3.491 17489673

[B35] Rector RS Warner SO Liu Y Hinton PS Sun GY Cox RH Stump CS Laughlin MH Dellsperger KC Thomas TR Exercise and diet induced weight loss improves measures of oxidative stress and insulin sensitivity in adults with characteristics of the metabolic syndrome Am J Physiol Endocrinol Metab 2007 293 2 E500 506 10.1152/ajpendo.00116.2007 17473052 PMC2646852

[B36] Roberts CK Won D Pruthi S Kurtovic S Sindhu RK Vaziri ND Barnard RJ Effect of a short-term diet and exercise intervention on oxidative stress, inflammation, MMP-9, and monocyte chemotactic activity in men with metabolic syndrome factors J Appl Physiol 2006 100 5 1657 1665 10.1152/japplphysiol.01292.2005 16357066

[B37] Reynolds K Lewis B Nolen JD Kinney GL Sathya B He J Alcohol consumption and risk of stroke: a meta-analysis JAMA 2003 289 5 579 588 10.1001/jama.289.5.579 12578491

[B38] Paffenbarger RS Hyde RT Wing AL Lee I-M Jung DL Kampert JB The association of changes in physical-activity level and other lifestyle characteristics with mortality among men New Engl J Med 1993 328 8 538 545 10.1056/NEJM199302253280804 8426621

[B39] Kottke TE Battista RN DeFriese GH Brekke ML Attributes of successful smoking cessation interventions in medical practice. A meta-analysis of 39 controlled trials JAMA 1988 259 19 2883 2889 10.1001/jama.259.19.2883 3367456

[B40] Hu FB Manson JE Stampfer MJ Colditz G Liu S Solomon CG Willett WC Diet, lifestyle, and the risk of type 2 diabetes mellitus in women N Engl J Med 2001 345 11 790 797 10.1056/NEJMoa010492 11556298

[B41] Esposito K Marfella R Ciotola M Di Palo C Giugliano F Giugliano G D’Armiento M D’Andrea F Giugliano D Effect of a mediterranean-style diet on endothelial dysfunction and markers of vascular inflammation in the metabolic syndrome: a randomized trial JAMA 2004 292 12 1440 1446 10.1001/jama.292.12.1440 15383514

[B42] Church TS Earnest CP Skinner JS Blair SN Effects of different doses of physical activity on cardiorespiratory fitness among sedentary, overweight or obese postmenopausal women with elevated blood pressure: a randomized controlled trial JAMA 2007 297 19 2081 2091 10.1001/jama.297.19.2081 17507344

[B43] Ford ES Giles WH Dietz WH Prevalence of the metabolic syndrome among US adults: findings from the third National Health and Nutrition Examination Survey JAMA 2002 287 3 356 359 10.1001/jama.287.3.356 11790215

[B44] Roberts LJ Morrow JD Measurement of F-2-isoprostanes as an index of oxidative stress in vivo Free Radical Biol Med 2000 28 4 505 513 10.1016/S0891-5849(99)00264-6 10719231

